# Species Diversity and Secondary Metabolites of *Sarcophyton*-Associated Marine Fungi

**DOI:** 10.3390/molecules26113227

**Published:** 2021-05-27

**Authors:** Yuanwei Liu, Kishneth Palaniveloo, Siti Aisyah Alias, Jaya Seelan Sathiya Seelan

**Affiliations:** 1 Institute of Ocean and Earth Sciences, Institute for Advanced Studies Building, University of Malaya, Kuala Lumpur 50603, Wilayah Persekutuan Kuala Lumpur, Malaysia; alec.liu2012@gmail.com (Y.L.); saa@um.edu.my (S.A.A.); 2 Institute for Tropical Biology and Conservation, Universiti Malaysia Sabah, Kota Kinabalu 88400, Sabah, Malaysia

**Keywords:** octocoral, marine fungi, holobiont, secondary metabolites, diversity

## Abstract

Soft corals are widely distributed across the globe, especially in the Indo-Pacific region, with *Sarcophyton* being one of the most abundant genera. To date, there have been 50 species of identified *Sarcophyton*. These soft corals host a diverse range of marine fungi, which produce chemically diverse, bioactive secondary metabolites as part of their symbiotic nature with the soft coral hosts. The most prolific groups of compounds are terpenoids and indole alkaloids. Annually, there are more bio-active compounds being isolated and characterised. Thus, the importance of the metabolite compilation is very much important for future reference. This paper compiles the diversity of *Sarcophyton* species and metabolites produced by their associated marine fungi, as well as the bioactivity of these identified compounds. A total of 88 metabolites of structural diversity are highlighted, indicating the huge potential these symbiotic relationships hold for future research.

## 1. Soft Corals

Soft corals, also known as octocorals, are Anthozoans (Ehrenberg, 1834) classified under the subclass Octocorallia (Haeckel, 1866). They belong to the Phylum Cnidaria, making them closely related to the sea anemones, hard corals and jellyfishes. Unlike hard corals that are the building blocks of the coral reef, soft corals act as shelter for juvenile fishes and food to some marine organisms. As the name octocoral is derived from Latin “octo”, which means eight, soft coral species comprise of eight-tentacle polyps and eight mesenteries, with minimal variance within the clade. The polyp in octocorals is an individual zooid, and they together play important roles in the essential functions of a colony, including growth, food capture, transport of nutrients, defence, irrigation of seawater and reproduction [1]. As suspension feeders, soft coral food intake relies on environmental conditions, especially water currents [2]. For small organic particles (<20 mm), octocoral polyps can filter them from the water column, whereas larger particles (such as zooplankton and larvae) could be captured or intercepted by the tentacles. Since octocorals have simple stinging cells (nematocysts), their food is restricted to weakly-swimming, small plankton [3].

Octocorals are widely distributed, with their presence recorded from the intertidal zone to depths up to 6400 m and from tropical to polar regions [4]. Their distribution is heavily influenced by several environmental factors, for example, distance from the coast, suspended organic matter and the presence of strong currents [5]. For instance, the distribution of cold-water species is closely related to salinity, temperature, productivity, oxygen, the broad scale of the highest diversity of soft corals in the world, of which are mostly endemic [6,7]. However, the greatest diversity of octocorals is recorded in the Indo-Pacific oceans [8], part of the Coral Triangle Region, which has the highest mega-biodiversity in the world. In fact, octocorals are particularly diverse in tropical, subtropical shallow reefs and deep-sea waters, often being dominant occupiers of the benthic community [9]. To date, up to 3500 octocoral species have been recorded and classified in up to 400 genera.

The subclass Octocorallia comprises three orders: Alcyonacea, Helioporacea and Pennatulacea. The order Alcyonacea (Lamouroux, 1816) has the most number of species among octocorals [1]. Alcyonacea consists of stoloniferous forms, soft corals, and gorgonians [10]. This classification scheme is currently followed by most taxonomists, even though Alcyonacea is still viewed as unstable together with many family-level classifications [9,11]. There are approximately 31 families of soft corals and sea fans under this order, despite the lack of defining synapomorphies [9]. Although this order has been divided into six sub-ordinal groups: Alcyoniina, Calcaxonia, Holaxonia, Protoalcyonaria, Scleraxonia and Stolonifera, there is no molecular analysis to support this classification scheme, which reflects morphological categories, not clades [1,9,11]. Most soft corals belong to the order Alcyonacea, which includes the families Xeniidae, Nephtheidae, and Alcyoniidae. The family Alcyoniidae consists of three genera *Lobophytum* (Marenzeller, 1886), *Sinularia* (May, 1898) and *Sarcophyton* (Lesson, 1834), and these genera are considered the most important contributors to the total biomass of Indo-Pacific reefs, which cover up to 25% of the reef surface [5].

Since the nineteenth century, soft corals have been the subjects of active biological research [12]. Soft corals are prolific producers of terpenoids of the cembranoid skeleton. However, due to the presence of symbiotic microorganisms in soft corals, there has been plenty of debate on the origin of the secondary metabolites of the hosts. This has given rise to investigations on the metabolites produced by coral symbiotic microorganisms. Since the 1980s, plenty of reviews have been compiled, documenting the metabolites produced by the coral-associated microorganisms. Based on the available compilation, we observed a high number of metabolites reported between 2000–2010, and a reduction thereafter. Most reviews report the metabolites produced by marine fungi and to date, there has been only one specifically on octocoral-associated microbes with reports from the span of 2006 to early 2016 with a focus on bioactivity [13]. This review reports metabolites produced by marine fungi isolated from the soft coral genus *Sarcophyton* compiled over a duration of 10 years from 2010–2020.

## 2. Diversity of *Sarcophyton*

In 1982, Verseveldt revised the classification scheme of *Sarcophyton* based on a systematic examination of the morphology and microscopic images of *Sarcophyton*-like specimens [14]. According to his revision on *Sarcophyton* taxonomy, the genus *Sarcophyton* contained 35 valid species, and since then, there have been reports on new species of *Sarcophyton*. To date, there have been approximately 50 *Sarcophyton* species as shown in Table 1. Most *Sarcophyton* species were identified in the Indo-Pacific regions.

## 3. *Sarcophyton*-Fungal Associations

Coral-associated microbes consist of endolithic algae, endosymbiotic dinoflagellates, bacteria, fungi, alveolates, archaea, and viruses. The consortium of coral and its associated internal and external microbes is often considered as the holobiont [43,44]. The associated microorganisms provide extra carbon and nitrogen sources for their host, as well as play a part in detoxification, nutrient cycling, genetic exchange, ultra violet (UV) protection, and chemical defence [43,45]. In some populations of gorgonian, fungal diseases are common, but relatively few investigations have been conducted on the causal marine fungi from these octocorals [43]. In particular, the genera *Aspergillus* and *Penicillium* have often been found in the Caribbean *Gorgonia ventalina* [46,47], *Leptogorgia* species distributed in the Eastern Pacific regions [48], and many octocorals in the South China Sea [49] as well as Singapore [50]. Other frequently identified octocoral-associated fungi include the genera *Cladosporium* [46,47,48], *Fusarium* [48,50,51], *Nigrospora* [48,51], and *Tritirachium* [46,47,48,50]. As for the soft coral genus *Sarcophyton*, *Aspergillus terreus* was obtained from the *Sarcophyton subviride*, which was collected from the coast of Xisha Island in the South China Sea [52]. The marine fungus *Penicillium bialowiezense* was also isolated from the same soft coral species [53]. Additionally, *Chondrostereum* sp. was isolated from *Sarcophyton tortuosum* of the South China Sea as well [54].

The surrounding environment and host substrate have an impact on the composition of fungal communities [51], with a varied abundance of the most common associated fungal species. Knowledge of the fungal isolates is mainly obtained from culture-based techniques, thus favouring species likely to be cultivated in the laboratory conditions [50,51]. Nonetheless, cultured marine fungi have been a promising reservoir of bioactive secondary metabolites, usually with unique chemical structures, thus making octocoral-derived fungi potential bio-prospecting sources [13]. Despite limited knowledge about the exact ecological functions of these fungal species, some possess potential antifungal, antibacterial properties and might have a role in maintaining holobiont health and regulating the microbiome [13]. Table 2 summarises the soft coral *Sarcophyton* and its associated fungi.

## 4. Metabolites of Marine Fungi Derived from *Sarcophytons*

Marine organisms are an important source of natural products with potential for drug discovery. To date, more than 40,000 marine natural products (MNPs) have been identified from the marine environment. Coral reefs are among the most productive ecosystems and exhibit a large group of structurally unique biosynthetic products [60]. The coral reef is a prolific source of metabolites synthesised by a wide range of organisms such as sponges, cnidarians, tunicates, molluscs, echinoderms, bryozoans, macroalgae and microorganisms. Interestingly, recent records have shown an upward trend in MNPs from marine microorganisms, with approximately 57% of the total metabolites reported in 2017 [60]. These MNPs can be classified into terpenoids, alkaloids, steroids, lactones, polyketides, peptides, phenols, and lipids based on their biosynthesis pathways. Most of these metabolites are of pharmaceutical interest due to the varying bioactivity exhibited, such as cytotoxic, antimicrobial, anti-inflammatory, antimalarial, and antidiabetic activities [61].

Due to the lack of calcium carbonate skeletons for physical protection, soft corals depend heavily on chemical defence mechanisms in order to resist predators and prevent overgrowth and fouling by accumulating a variety of secondary metabolites in their bodies and releasing them to the environment [62]. The soft coral genus *Sarcophyton* hosts a wide diversity of marine fungi that interacts with the soft corals in multiple ways. The microorganisms are expected to synthesise various secondary metabolites to adapt and survive in their cohabitating environment either as a symbiont or as a parasite [62]. The fungal genus *Aspergillus*, for instance, was once thought to be pathogenic; however, it was not only found in diseased gorgonians but also healthy ones [46]. Therefore, it is now considered as an opportunist rather than a pathogen. Soft coral-associated fungi have an influence on the maintenance of holobiont health and regulation of the microbiome. Previous studies have demonstrated that coral-associated bacteria communities regulate the settlement of bacteria on the coral surface, thus controlling the resistance against coral disease [63]. The protective mechanisms include competition for food and space, as well as the production of antibiotics from the mucus or coral tissues [45]. Although corals naturally produce a mucus microbiome as a defence system against pathogens [64], changes in the microbiome could lead to the emergence of coral diseases. However, associated bacterial communities produce antibiotic metabolites to inhibit the settlement and growth of many pathogenic species, like *Vibrio coralliilyticus*, *V. shiloi* and *Serratia marcescens* [45]. Even though there is no such study carried out on associated fungi, they could play a similar role to coral-associated bacteria.

The conventional view of microbial symbionts has been that their biosynthesis of natural products contributes greatly to the wide range of metabolites from sessile marine invertebrates. In the case of sponges, there have been debates on the source of metabolites from this organism. Eventually, it was determined that the microorganisms within are the main contributors of secondary metabolites [65]. In the case of hard corals, the production of mycosporin amino acids (MAA) provides protection for the corals against solar radiation [66]. Similarly to the relationship between fungi and soft corals, the metabolites produced by the fungi are of interest. The bioactivity exhibited can be associated with the protective role of the soft corals. Additionally, this work also confirms that the metabolites produced by the fungi are totally different from those reported from the soft corals, ascertaining that the soft coral metabolites are synthesised by the coral itself. Even though most octocoral-derived marine fungi are obtained through cultivation-dependent methods, these fungi produce a variety of bioactive natural products, usually exhibiting an unusual chemical structure [13]. Thus, octocoral-derived fungi provide a great candidate for bio-prospecting. The following sections compile the soft coral-fungal associated metabolites that have been reported over the years 2010–2020. Most of the secondary metabolites belong to the chemical sgroup sesquiterpene and indole alkaloid. A list of compounds with bio-activity is shown in Table 3.

### 4.1. Terpenoids

#### Sesquiterpene

Soft-coral associated fungi are reported to be an important source of sesquiterpenes. The earliest reports on sesquiterpenes from soft-coral associated fungi are the isolation and characterisation of the hirsutane sesquiterpenes hirsutanol A (**1**), E (**2**) and F (**3**) in 2011 from the marine fungus *Chondrostereum* sp., which was isolated from the soft coral *Sarcophyton tortuosum* [67]. The laboratory-cultured fungal isolate was extracted in over ethyl acetate prior to fractionation using petroleum ether (Petr Eth), ethyl acetate (EtOAc) and methanol (MeOH) as a mobile phase. A 60% gradient reverse phased high-performance liquid chromatography (RP-HPLC) profiling led to the isolation of the hirsutanols A (**1**), E (**2**) and F (**3**). Initial reports on hirsutanol A (**1**) were from the marine sponge *Jaspis* cf. *johnstoni* fungal isolate in 1986, which was also isolated from an unidentified fungal strain from the *Haliclona* sponge along with hirsutanol F (**3**) [73]. Hirsutanol E (**2**) (C_l5_H_24_O_3_) comprises of three methyls, five methylenes, two methines, five quaternary carbons, and three hydroxy groups. According to nuclear magnetic resonance (NMR) and single-crystal X-ray diffraction data, the structure of hirsutanol F (**3**) was regarded the same as gloeosteretriol, despite the opposite optical rotations [67]. Hirsutanol A was characterised as C_l5_H_18_O_3_ with potential cytotoxicity against many types of human cancer cell lines and induction of autophagical cell death through increased Reactive Oxygen Species (ROS) levels. An investigation into the anticancer mechanism of hirsutanol A (**1**) towards MCF-7 breast cancer cells exhibited the inhibition of cell proliferation, enhanced ROS production, apoptosis and autophagy. Hirsutanol A (**1**) could lead to apoptosis and autophagy through accumulated ROS production, and MCF-7 cells could be sensitised if co-treated with an autophagy inhibitor [74]. The bioactivity of hirsutanol A (**1**) was attributed to the presence of an α-methylidene oxo group, which was absent in hirsutanols E (**2**) and F (**3**) [67].

In 2012, an additional hirsutane type sesquiterpene, hirsutanol C (**4**) was isolated along with five triquinane-type sesquiterpenoids, chondrosterins A–E (**5**–**9**) from the fungus *Chondrostereum* sp. isolated from tissues of *Sarcophyton tortuosum* [54]. The isolated fungi were laboratory-cultured in potato dextrose broth (PDB) medium that was eventually extracted over EtOAc. A two-stage column chromatography fractionation with Petr Eth/EtOAc followed by EtOAc/MeOH and an RP-HPLC purification with a 60–100% acetonitrile (MeCN) gradient system through a Shim-Pack Octadecylsilyl (ODS) column (250 × 20 mm) yielded hirsutanol C (**4**). Subsequent Sephadex LH-20 gel column chromatography and RP-HPLC purification of the fungal fractions yielded chondrosterins A–E (**5**–**9**) [54]. Hirsutanol C (**4**), C_15_H_20_O_3_, isolated as powder, was previously characterised by Wang et al. (1998) [73] from an unidentified fungus of the marine sponge *Haliclona* sp. that yielded the hirsutanols A (**1**) and F (**3**) [54]. The relative configuration of hirsutanol C (**4**) was determined via single-crystal X-ray diffraction. It was inactive against the human lung cancer cell line (A549), human nasopharyngeal carcinoma cell line (CNE2), and human colon cancer cell line (LoVo) at IC_50_ (half maximal inhibition concentration) concentrations >200 μM [54]. Chondrosterins A (**5**) and B (**6**) were both isolated as yellowish oil. With the presence of a α-methylene ketone group in its tricyclic system, chondrosterin A (**5**) showed significant cytotoxic activities against various cancer lines A549 (IC_50_ = 2.45 μM), CNE2 (IC_50_ = 4.95 μM), and LoVo (IC_50_ = 5.47 μM) [54]. The metabolites from *Chondrostereum* sp. cultured in PDB medium showed a difference from those in the glucose peptone yeast (GPY) medium. This investigation also evaluated the difference in metabolite presence through alteration of the fermentation conditions, such as the ratios of the carbon and nitrogen source and inorganic salts, leading to the detection of the previously reported hirsutanol E (**2**) in the GPY culture strain. This confirms that the *Chondrostereum* sp. is able to produce diverse hirsutane derivatives under different conditions [54]

Chondrosterin C (**7**) is a compound with a hydroxyl, ketone carbonyl, α,β-unsaturated carbonyl functionality, and its planar skeleton is determined entirely by ^1^H-^1^H correlated spectroscopy (COSY) and Heteronuclear Multiple Bond Correlation (HMBC) analysis [54]. Chondrosterin D (**8**) was isolated as a colourless crystal. Similar to chondrosterin C (**7**), this compound also possesses three ketone carbonyl groups. Infrared absorptions at 1737, 1687 and 1610 cm^−1^ confirmed the presence of ketones and α,β-unsaturated carbonyls. X-ray crystallography was used to confirm its relative configuration [54]. The fifth compound from the cultured *Chondrostereum* sp. is chondrosterin E (**9**), which was reported as a white solid. Compared to the other metabolites isolated from this study, chondrosterin E (**9**) is the only compound where the carbonyl was positioned at C-5 instead of C-4 [54].

In a separate study, following the successful characterisation of chondrosterins **A**–**E** (**5**–**9**), additional hirsutane sesquiterpenoids chondrosterin F (**10**), incarnal (**11**) and anthrosporone (**12**) were reported from the soft coral species *Sarcophyton tortuosum*-associated marine fungus *Chondrostereum* sp. collected from South China Sea in 2013 [68]. Using the similar culture and isolation protocol involving two stages of column chromatography followed by RP-HPLC purification as the previously mentioned metabolites, chondrosterin F (**10**) was isolated in the form of a colourless oil. This compound was determined to have a rearranged hirsutane skeleton believed to be caused by the migration of a methyl functionality from C-2 to C-3 as well as the formation of a lactone through the conversion of a cyclic ketone [68].

The hirsutane incarnal (**11**), a compound previously first reported from fungus *Gloeostereum incarnatum*, was isolated as red solids from the soft-coral-associated *Chondrostereum* sp. [68]. Compared to the reference data, the *J* value coupling constant of the protons H-11α and H-11β reported in this study was calculated as 14.0 Hz instead of an oddly lower value of 5.1 Hz that was previously reported. Incarnal (**11**) demonstrated potent cytotoxic effects on various cancer cell lines, including LoVo (IC_50_ = 2.16 μg mL^−1^), CNE2 (IC_50_ = 6.07 μg mL^−1^), A549 (IC_50_ = 12.37 μg mL^−1^), human nasopharyngeal carcinoma cell line (SUNE1) (IC_50_ = 3.99 μg mL^−1^), human breast cancer cell line (MCF-7) (IC_50_ = 4.57 μg mL^−1^), human nasopharyngeal carcinoma cell line (CNE1) (IC_50_ = 8.33 μg mL^−1^), human hepatic cancer cell line (Bel7402) (IC_50_ = 23.36 μg mL^−1^), human epidermoid carcinoma cell line (KB) (IC_50_ = 28.55 μg mL^−1^) [68]. Based on these data, it is evident that the α-methylene ketone functional group plays an important role in the cytotoxic activities of hirsutane sesquiterpenoids. Arthrosporone (**12**) is another hirsutane sesquiterpenoid originally reported from an unidentified arthroconidial fungus and *Macrocystidia cucumis* [75]. In comparison with chemical data from the literature, it was confirmed that arthrosporone (**12**) was also produced by the investigated soft-coral-associated fungi. Arthrosporone (**12**) was not reactive in oxidation reactions, which is a common characteristic of the tertiary hydroxyl groups present in the compound [68].

In 2014, two more hirsutane sesquiterpenoids, chondrosterins I and J (**13** and **14**), were obtained from yet again the marine fungus *Chondrostereum* sp., which originated from *Sarcophyton tortuosum* and cultured in a liquid medium with glycerol as the carbon source [69]. The compounds were isolated through repeated Petr Eth/EtOAc and EtOAc/MeOH column chromatography on the EtOAc extract followed by RP-HPLC purification. Though cultured in a different medium, the fungal extract contained the previously reported hirsutanol A (**1**), chondrosterins A (**5**) and incarnal (**11**). Compared to the previously mentioned hirsutane sesquiterpenoids, chondrosterins I (**13**) and J and (**14**) exhibited a switch in methyl position from C-2 to C-6 and a presence of carboxylated methyl at C-3 [69]. Chondrosterin I (**13**) was isolated as a colourless solid. The absolute configuration of chondrosterin I (**13**) was determined as 1*R*, 6*S*, 8*S* and confirmed by X-ray single-crystal diffraction. Chondrosterin J (**14**) was isolated as a white solid. The absolute configuration for this compound was established as 1*R*, 6*S*, 7*S*, 8*S*. These compounds were screened for cytotoxicity against the human nasopharyngeal cancer cell line CNE-1 and CNE-2, where chondrosterin J (**14**) was cytotoxic against CNE-1 and CNE-2 cell lines with the IC_50_ values of 1.32 and 0.56 μM [69].

The mycelia of *Chondrostereum* sp. of *Sarcophyton tortuosum* cultured in GPY liquid medium was reported to contain four sesquiterpenoids, which included three triquinane-type sesquiterpenoids, chondrosterins K–M (**15**–**17**) and a previously identified metabolite, anhydroarthrosporone (**18**) [70]. A similar fractionation and purification technique was applied in order to isolate and characterise the compounds **15**–**18**. The use of GPY medium often results in an altered metabolite profile compared to those cultured in PDB medium. Chondrosterin K (**15**), C_15_H_22_O_3_ was obtained in the form of a colourless oil, with five degrees of unsaturations. [70]. In contrast, chondrosterin L (**16**) lacks two methines and possessed seven quaternary carbons compared to chondrosterin K (**15**) [70]. Chondrosterins L (**16**) and M (**17**) from this fungal extract are hirsutanes with almost identical functional groups. Both the chondrosterins were obtained in the form of yellowish oil. Structurally, chondrosterin L (**16**) differed from chondrosterin M (**17**) due to the presence of an exo-methylene at carbon position 3 instead of the secondary methyl, CH_3_CH– functionality that was found in chondrosterin M (**17**) [70]. The fourth compound reported from this study was anhydroarthrosporone (**18**), a hirsutane sesquiterpene that was initially isolated from the fungus *Ceratocystis ulmi* extract [76]. Huang et al. (2016) reported it for the first time from soft-coral-derived fungi. Anhydroarthrosporone (**18**) is a metabolite that contained a β-substituted α,β-unsaturated cyclopentenone. The anhydroarthrosporone (**18**) is a derivative of the previously reported arthrosporone (**12**) with the presence of a double bond between carbons C-5 and C-6 [68]. Chondrosterins K-M (**15-17**) demonstrated significant *in vitro* cytotoxicity against seven cancer cell lines; CNE1, CNE2, SUNE1, A549, epithelial tumour cell line (HONE1), Gejiu Lung Carcinoma-82 (GLC-82) and normal human liver cell (HL7702) [70].

**Figure 1:**
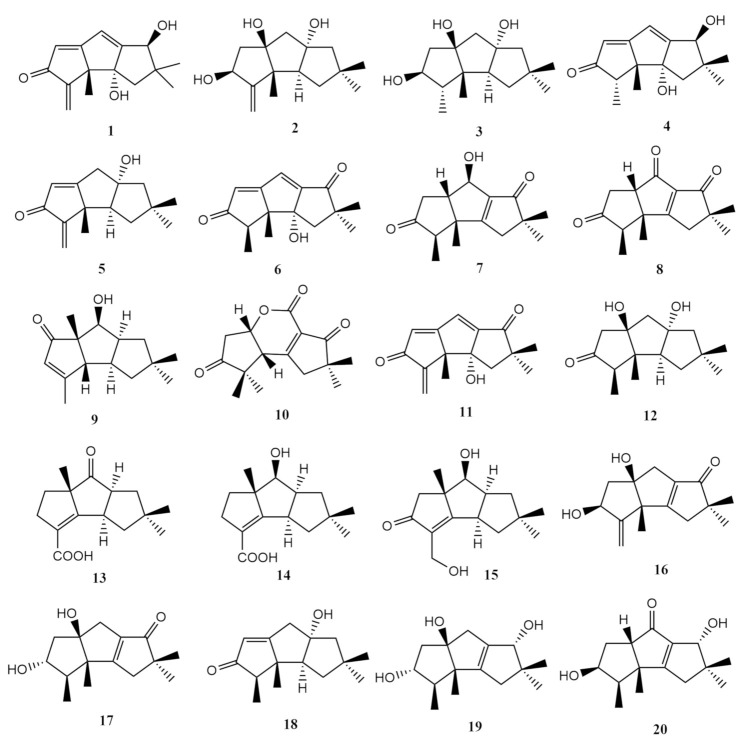
Hirsutanols from *Sarcophyton*-associated fungi.

Further investigation into the *Sarcophyton tortuosum*-derived fungi *Chondrostereum* sp. cultivated in GPY medium continued to yield two more additional hirsutane-type sesquiterpenoids, chondrosterins N (**19**) and O (**20**) [55]. These were isolated from its EtOAc extract after repeated column chromatography followed by RP-HPLC over a 70% MeCN mobile phase [55]. Both chondrosterins N (**19**) and O (**20**) were isolated as colourless oil. Chondrosterin N (**19**) is comprised of an α, β-unsaturated carbonyl chromophore as indicated by UV absorption at 239 nm. On the other hand, chondrosterin O (**20**) was identified as a stereoisomer to chondrosterin N (**19**). Both compounds were initially determined as identical based on ^1^H–^1^H COSY and HMBC spectra; however, the differences in the chemical shifts of H-4 and its coupling constants were able to distinguish these compounds from each other [55]. They were screened against seven cancer cell lines: CNE1, CNE2, HONE1, SUNE1, A549, GLC82 and HL7702, and were categorised as inactive with IC_50_ values exceeding 100 μM. Chemical structures of all the highlighted I-associated fungi hirsutane sesquiterpenes are exhibited in Figure 1.

### 4.2. Alkaloids

#### Indole Type Alkaloid

Indoles are bicyclic molecules built by a six-membered benzene ring fused to a five-membered pyrrole ring. These compounds are commonly produced by a wide variety of microorganisms. As for the *Sarcophyton* associated fungi, in 2013, two cytochalasin compounds, aspochalasin A1 (**21**) and cytochalasin Z24 (**22**), were reported from a *Sarcophyton* sp.-derived marine fungi *Aspergillus elegans*, originating from the South China Sea [58]. The alkaloids reported in this study were isolated from the EtOAc extract that was subjected to a petroleum ether/EtOAc followed by a chloroform (CHCl_3_) fractionation over Sephadec LH-20 [58]. The reported compounds were purified over HPLC using an ODS Kromasil C18 column with the mobile phase between 60 to 85% MeOH. Aspochalasins are a subgroup of cytochalasans, consisting of a macrocyclic ring, isoindolone moiety and a 2-methyl-propyl side chain. According to high-resolution electrospray ionisation mass spectrometry (HRESIMS), aspochalasin A1 (**21**), isolated as a white powder, was characterised as C_24_H_35_NO_5_. The presence of a (2-methylpropyl) isoindolone moiety is an indication of aspochalasin A1 (**21**) from a typical cytochalasin skeleton [58]. Cytochalasin Z24 (**22**) was also isolated as a white powder and possesses a 10-phenyl-substituted 6,7-epoxyperhydroisoindol-1-one type skeleton. Both compounds, aspochalasin A1 (**21**) and cytochalasin Z24 (**22**), were determined to share identical macrocyclic properties similar to other known cytochalasins reported. The absolute configuration of cytochalasin Z24 (**22**) was determined as 3*S*,4*S*,5*S*,6*R*,7*S*,8*S*,9*S*,13*E*,16*S*,18*S*,19*E* [58].

Eight additional cytochalasin-derivatives (**23**–**30**) were also reported from the *Aspergillus elegans* [58]. All these cytochalsins were previously reported from various sources of fungi from the genus *Aspergillus*. Aspochalasins B (**23**), a yellowish powder and D (**24**), were previously reported from the *Aspergillus niveus* that was associated with a marine crustacean [77], while aspochalasin H (**25**) was first identified from the aspochalasin D-producing strain, *Aspergillus* sp. The common detection of a broad infra-red band at 1685 cm^−1^ shows the presence of lactone and ketone carbonyl in aspochalasins B (**23**) and D (**24**). The carbon position C-18 of aspochalasin D (**24**) was attached to a hydroxyl moeity instead of a carbonyl as in aspochalasin B (**23**). Aspochalasin H (**25**), C_24_H_35_NO_5_ was isolated as a colourless powder [78] and was reported to have an identical stereochemistry to aspochalasin D (**24**). Additionally, the double bond between carbons C-19 and 20 was replaced by an epoxy in aspochalasin H (**25**), which differed both these compounds structurally. Aspochalasins I (**26**) and J (**27**) were obtained from *Aspergillus flavipes* associated with the rhizosphere of *Ericameria laricifolia*, a turpentine bush [79]. Aspochalasin I (**26**) was isolated as a white powder [79], while aspochalasin J (**27**) was isolated as a white solid. The difference between aspochalasins (**26**) and J (**27**) was the presence of only one oxygenated methine bearing a hydroxyl with α-orientation in compound **27** [79]. The acetylation of aspochalasin J (**27**) yields the monoacetyl derivative acetyl aspochalasin J confirming the presence of hydroxyl (-OH) in the compound.

Aspergillin PZ (**28**) is a compound that was previously reported from the soil fungi *Aspergillus awamori* as a colourless crystal [80]. Aspergillin PZ (**28**) shares an identical skeleton to the compound aspochalasin C, which is also produced by fungi from the genus *Aspergillus*. The addition of a hydroxyl followed by cyclisation of aspochalasin C produced aspergillin PZ (**28**). Zygosporin D (**29**) was previously isolated from the fungus *Metarrhizium anisopliae* [81], while rosellichalasin (**30**), a solid colourless needle, has been reported from an *Aspergillus* strain from China [82]. Zygosporin D (**29**) is reported as a deacetyl derivative of the compound cytochalasin D. These compounds are classified as cytochalasins, a group of fungal alkaloids with diverse biological activities targeting cytoskeletal processes. They can bind to actin filaments and block polymerisation and the elongation of actin. The isolated compounds were screened for their bio-activity against six terrestrial pathogenic bacteria (*Staphylococcus epidermidis*, *S. aureus*, *Escherichia coli*, *Bacillus subtilis*, *B. cereus* and *Micrococcus luteus*) and two marine pathogenic bacteria (*Vibrio parahaemolyticus* and *Listonella anguillarum*) [58].

**Figure 2:**
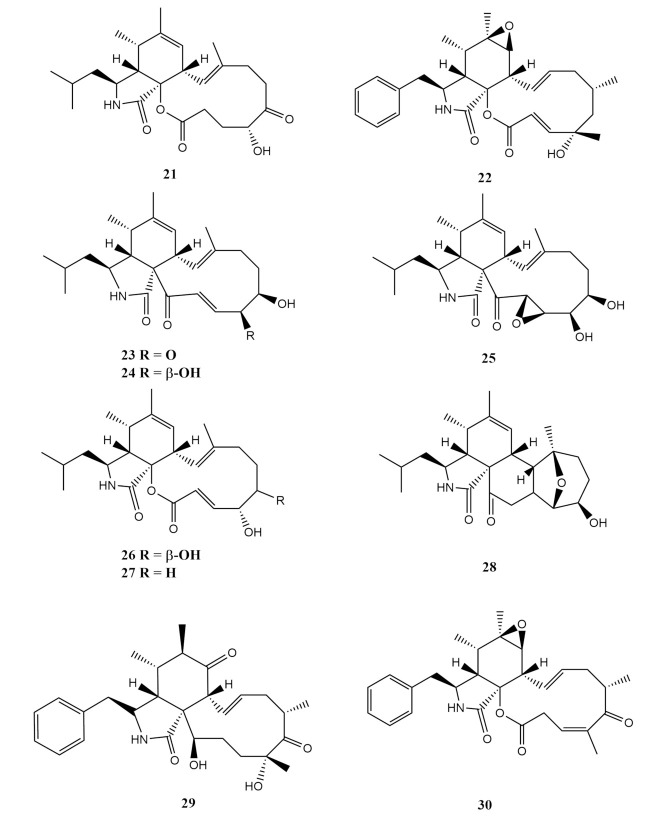
The chemical structures of the types of indole alkaloids isolated from the soft-coral-associated fungi.

Aspochalasin D (**24**) demonstrated a wide spectrum of antibacterial properties, especially towards four pathogenic bacteria, *S. epidermidis*, *S. aureus*, *E. coli* and *B. cereus* [58]. In contrast, aspochalasin I (**26**) displayed moderate minimum inhibition activity (MIC) against the bacteria *S. epidermidis* (MIC = 20 μM) and *S. aureus* (MIC = 10 μM). In addition, compounds aspochalasin D, H-J (**24**–**27**) also showed strong antifouling activity against the larval settlement of the barnacle, *Balanus amphitrite*, with EC_50_ values of 6.2, 37, 34 and 14, respectively [58]. Despite the small differences in their structures, aspochalasin D (**24**), which possessed an α,β-unsaturated lactone moiety, demonstrated that the electrophilic α,β-unsaturated carbonyl moiety plays an important role in the antifouling activity of these cytochalasins [58]. Since aspochalasin D (**24**) had higher antifouling activity than aspochalasin H (**25**), the presence of a double-bond at C-19 and C-20 was deduced to be the possible active site for cytochalasin antifouling activities [58]. The chemical structures of these compounds are shown in Figure 2.

A total of 13 additional indole alkaloids were also isolated from the *Saracophyton* sp. associated marine fungus *Pseudallescheria boydii* from the South China Sea [59]. These compounds comprised the two bisindoles, pseudboindoles A (**31**) and B (**32**). The other metabolites characterised were 3,3^′^-cyclohexylidenebis(1H-indole) (**33**), 3,3-bis(3-indolyl)butane-2-one (**34**), 2-[2,2-di(1H-indol-3-yl) ethyl] aniline (**35**), 3,3^′^-diindolyl(phenyl) methane (**36**), 1,1-(3,3^′^-diindolyl)-2-phenylethane (**37**), perlolyrin (**38**), pityriacitrin (**39**), 1-acetyl-β-carboline (**40**), 3-hydroxy-β-carboline (**41**), 1-(9H-pyrido[3,4-b]indol-1-yl)ethan-1-ol (**42**) and N_b_-acetyltryptamine (**43**). These compounds were isolated from the EtOAc extract of the laboratory-cultured strain after fractionation over Petr Eth and EtOAc. The compounds were purified using HPLC and a Capcell-Pak C18 UG80 (250 × 20 mm) column with methanol and purified water as the mobile phase [59].

Pseudoindole A (**31**) was isolated as an amorphous brown powder, consisting of one methylene group, six methine groups, three quaternary carbon atoms and is built of two identical structural moieties comprising an ortho-disubstituted aromatic ring and 3-substituted indole connected at the electronegative carbon C-9 [59]. Chemically, pseudoindole A (**31**) was deduced as 1,3-di(1H-indol-3-yl)propan-2-ol. Pseudoindole B (**32**) is also made up of a similarly identical skeleton connected at the methine carbon C-8, which bears a chain with a sulfoxide moiety. Compounds **33** to **37** share a common basic structure comprising two identical groups of ortho-disubstituted aromatic ring and 3-substituted indole joined at carbon C-8, making them a member of the bisindole alkaloid class. Similar to compound **31** and **32**, 3,3^′^-cyclohexylidenebis(1H-indole) (**33**) was also isolated as a brown amorphous powder [59]. It can be synthesised by reacting an indole with cyclohexanone. This compound exhibited a 140% enhancing potential towards the Am80-induced HL-60 (myeloid leukemic cell lines) cell. When treated with eight human cancer cell lines (A549, GLC82, CNE1, CNE2, HONE1, SUNE1, BEL7402 and the human hepatocarcinoma cell line (SMMC7721)), compound **33** showed cytotoxicity with IC_50_ values of 22.84, 22.04, 18.69, 20.84, 26.62, 20.54, 27.52 and 22.50 μM, respectively [59]. 3,3-bis(3-indolyl)butane-2-one (**34**) was previously reported as a synthesised product but was later isolated as a natural metabolite from the bacterium *Vibrio parahaemolyticus* of the North Sea, as a pale yellowish solid [83]. Another indole reported from the soft-coral-derived fungi is 2-[2,2-di(1H-indol-3-yl) ethyl] aniline (**35**), which was previously isolated from the bacterium *Aeromonas* sp. derived from seawater collected from the South China Sea [84]. It is also a common product produced by various other bacterial sources and exhibits weak toxicity against the A549 cell line with an IC_50_ value of 22.6 μM [84].

The compounds 3,3^′^-diindolyl(phenyl)-methane (**36**) and 1,1-(3,3^′^-diindolyl)-2- phenylethane (**37**) were reported from the bacteria *Edwardsiella tarda* [85]. 3,3^′^-diindolyl (phenyl)-methane (**36**) was obtained as a red solid, while 1,1-(3,3^′^-diindolyl)-2-phenylethane (**37**) was reported as a yellowish solid. The compound 3,3^′^-diindolyl(phenyl)methane (**36**) was found to exhibit weak antibacterial properties against the pathogen *Clostridium perfringens* [86]. Perlolyrin (**38**), a type of β-carboline derivative isolated as a yellow powder, was originally reported as a fluorescent compound from soy sauce. It is also often associated with plants, such as Ginseng and several Asiatic plants. These derivatives are strongly associated with its antitumour and anti-oxidative properties [67]. Pityriacitrin (**39**), on the other hand, was previously reported from the marine bacterium *Paracoccus* sp. and the yeast *Malassezia furfur* [87]. It appeared as a bright yellow band in thin layer chromatography and was isolated as a yellow solid. Pityriacitrin (**39**) was characterised as a natural UV filter in cultures of the yeast *Malassezia furfur* [88].

**Figure 3:**
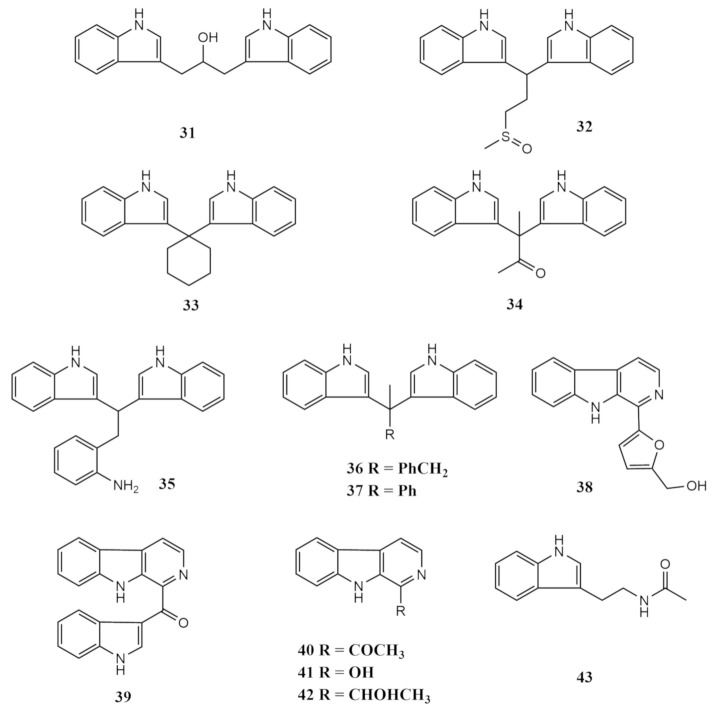
The chemical structures of the indoles of type isolated metabolites.

Along with the bisindole alkaloids, several β-carboline type indoles were also reported from the soft-coral associated *Pseudallescheria boydii*. 1-acetyl-β-carboline (**40**) is a fluorescent compound first reported from the marine sponge *Tedania ignis* [59]. Prior records of this metabolite were from a terrestrial plant *Ailanthus malabarica* [67]. Similarly, the other derivative is 3-Hydroxy-β-carboline (**41**), which was first obtained in its natural form as a yellowish amorphous solid from the stems of a medicinal plant *Picrasma quassioides* collected in China. This carboline derivative was previously described as a synthesised product [89]. The final two indoles reported from the investigation of Yuan and team (2019) [59] were 1-(9H-pyrido[3,4-b]indol-1-yl)ethan-1-ol (**42**), initially reported from the heartwood of *Dicorynia guianensis* as a yellowish powder [90] and N_b_-acetyltryptamine (**43**), which was isolated from an unidentified marine fungus derived from the red alga *Gracilaria verrucose* as a yellowish oil. Previous records on N_b_-acetyltryptamine had been as a bio-transformed product of tryptamine from the fungus *Streptomyces staurosporeus* [91]. All the chemical structures of these compounds are shown in Figure 3.

### 4.3. Anthraquinones Derivates

Anthraquinones are aromatic compounds with the 9,10-anthracenedione core and are often referred to as 9,10-dioxoanthracene with a keto functionality in its central ring. There have been nearly 100 naturally occurring anthraquinones, and about 20 have been identified to be the products of marine fungi derived from the soft-coral genus *Sarcophyton*. In 2012, Zheng et al. isolated tetrahydroaltersolanol B (**44**), five hydroanthraquinone derivatives named tetrahydroaltersolanols C–F (**45**–**48**) and dihydroaltersolanol A (**49**), from the liquid culture of *Alternaria* sp. derived from a *Sarcophyton* sp. collected from the Weizhou coral reef in the South China Sea [49]. The crude extract of the cultured fungi was fractioned using column chromatography with the mobile phase combination between Petr Eth:EtOAc (1:2). The immunosuppressive potential of the fractions exhibited activity at concentrations 1.48 ± 0.15 and 11.83 ± 0.83 μg/mL [49]. Repeated Sephadex column chromatography followed by HPLC over methanol through a Zorbax SB-C18 (9.4 mm × 25 cm) yielded the above-mentioned metabolites.

Tetrahydroaltersolanol B (**44**), isolated as a colourless crystal, is a hexahydroanthronol type anthraquinone isolated only from the fungi *Alternaria solani* [49]. So far there have been two records of this metabolite from the fungi. Likewise, tetrahydroaltersolanol C (**45**) was also isolated as a colourless crystal. According to the spectroscopic features, compound **45** bears a great deal of structural similarity to the compound tetrahydroaltersolanol B (**44**) with differences in the α, β positioning of the proton and hydroxyl at carbon C-3 and C-9, respectively [49]. Tetrahydroaltersolanol C (**45**) was isolated as a new metabolite at the point of report, along with tetrahydroaltersolanols D–F (**46**–**48**) from the soft-coral associated *Alternaria* sp. It exhibited antiviral activity when screened against the porcine reproductive and respiratory syndrome virus (PRRSV) [49].

Tetrahydroaltersolanol D (**46**) was also obtained in the form of colourless crystal. Though tetrahydroaltersolanol D (**46**) is structurally identical to tetrahydroaltersolanol B (**44**), it varied stereochemically at carbon positions C-1a and C-4a [49]. The relative configurations of all asymmetric carbons in tetrahydroaltersolanol D (**46**) were confirmed as 1aβ, 3β, 4aα, 9β, and 11β, identical to those of tetrahydroaltersolanol B (44). Likewise, tetrahydroaltersolanol E (**4**7) was isolated as a colourless crystal of similar nature to compounds (**45**) and (**46**). Since its chemical shifts resembled tetrahydroaltersolanol B (**4**4), it was eventually determined as 3-epi-tetrahydroaltersolanol B [49]. Tetrahydroaltersolanol F (**48**) was isolated as amorphous pink powder. It shows close structural resemblance to tetrahydroaltersolanol B (**44**), despite obvious differences in the presence of a singlet methyl at 2.14 ppm and the downfield shift of H-3 in ^1^H-NMR. The final compound in the set of hydroanthraquinones reported from the *Alternaria* sp. was dihydroaltersolanol A (**49**), isolated as colourless crystals as well [49]. The relative configurations of all asymmetric carbons in dihydroaltersolanol A (**49**) were determined as 1α, 1aα, 3β, 9β, and 11β. None of these compounds exhibited antimicrobial potential as screened [49].

Further investigation of the marine fungi *Alternaria* sp. from the *Sarcophyton* soft coral yielded six alterporriol-type anthranoid dimers, altersolanols B–C (**50**–51), altersolanol L (**52**), ampelanol (**53**), macrosporin (**54**) and alterporriol C (**55**), together with five more analogues, alterporriols N–R (**56**–**60**) were isolated and characterised [49]. The *Alternaria* sp. isolated from the soft coral was cultured in potato glucose liquid medium before being extracted in EtOAc. Column fractions of extract were subjected to repeated column chromatography over Sephadex LH-20 and purification via HPLC using a Kromasil C18 preparative HPLC column to yield the reported compounds [49].

**Figure 4:**
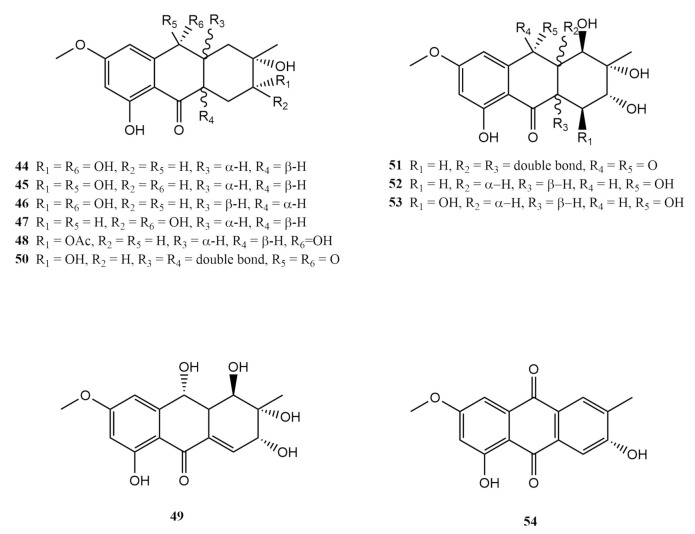
The chemical structures of compounds **44**–**54**.

Altersolanol B (**50**), a red needle and altersolanol C (**51**) were previously reported from the extract of *Alternaria solani*, which caused the black spot disease. Both compounds were potent in inhibiting all the Gram-positive bacteria [71]. Altersolanol L (**52**), isolated as a brown powder, and the white crystal macrosporin (**54**) reported from the soft coral *Alternaria* sp. were initially isolated from the endophytic fungus *Stemphylium globuliferum* derived from a medicinal plant species [92]. Apart from *Alternaria*, macrosporin (**54**) is known to be produced by several economically important crop-disease-causing fungal pathogens, such as *Cladosporium*, *Dichotomophthora*, *Phomopsis*, *Stemphylium* and *Dactylaria*. Altersolanol L (**52**) was reported to share a similar skeletal structure to the previously described dihydroaltersolanol A (**49**). Ampelanol (**53**), on the other hand, is another metabolite associated with medicinal plant-derived fungus *Ampelomyces* sp. It was isolated as white crystals and determined to exhibit mild cytotoxicity towards mouse lymphoma cells (L5178Y) [72]. The chemical structures of the anthraquinones compounds **44**–**54** are shown in Figure 4.

**Figure 5:**
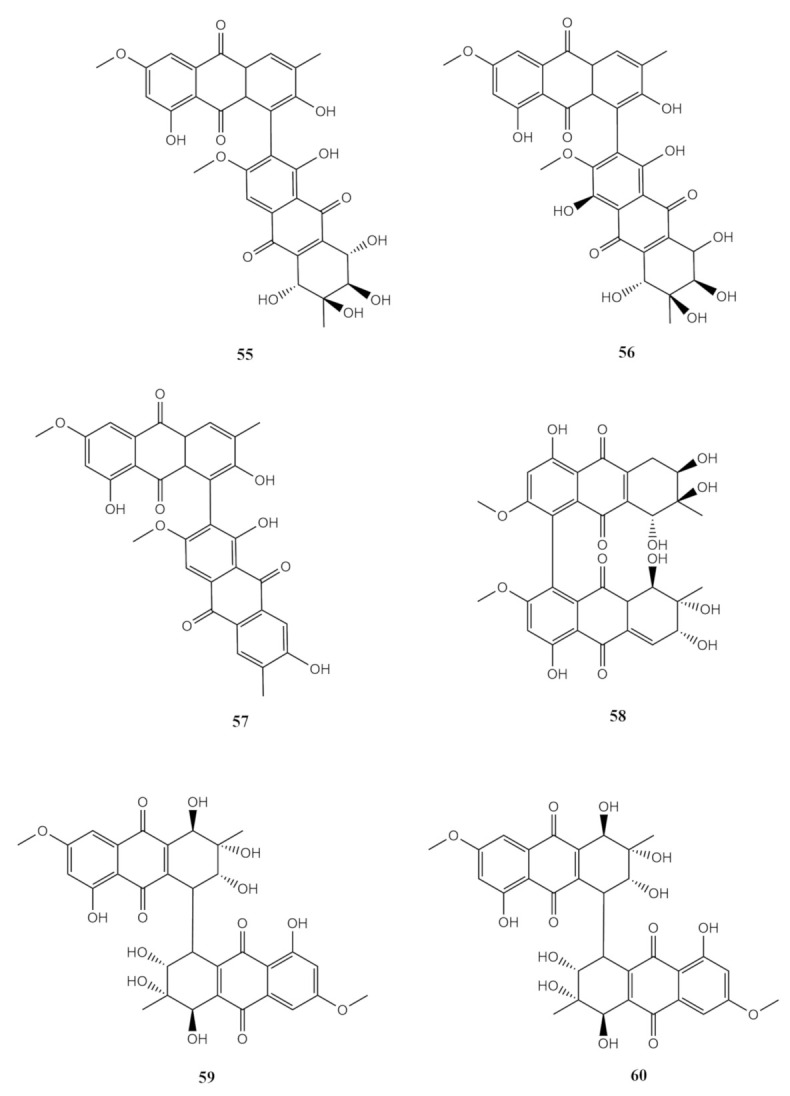
The chemical structures of the reported bianthraquinones.

Subsequently, several bianthraquinones were isolated from the soft-coral-derived marine fungi as well. Alterporriol C (**55**) belongs to a modified bianthraquinone and was first isolated from the fungus *Alternaria porri* as red needles [93]. Alterporriol C (**55**), which was antibacterial against *Escherichia coli* and *Vibrio parahemolyticus* with both MIC values 2.5 μM, is suggested to be made up of the compounds altersolanol A and macrosporin (**54**) [49]. Alterporriol N (**56**) was an amorphous powder in red. ^13^C NMR spectrum analysis suggested that alterporriol N (**56**) was a symmetrical dimer of altersolanol C (**51**) with a C-8 and C-8^′^ linkage [49]. Another symmetrical dimer to altersolanol C (**51**) isolated from *Alternaria* sp. was alterporriol O (**57**), which appeared as a red amorphous powder. Unlike alterporriol N (**56**), alterporriol O (**57**) was an anthranoid dimer with a C-4 and C4’ linkage [49]. Likewise, alterporriol P (**58**) was also a red, amorphous powder, with the molecular formula of C_32_H_26_O_12_. Alterporriol P (**58**), also isolated as a red amorphous powder, was characterised as a sub-unit of the altersolanol C (**51**) and macrosporin (**54**) linkage via carbon C-4 and C-6’. This compound was cytotoxic against the human prostate cancer (PC-3) and human colorectal carcinoma (HCT-116) cell lines with the IC_50_ values 6.4 and 8.6 μM [49]. In contrast, alterporriol Q (**59**) was obtained as a yellowish amorphous powder, and the final anthraquinone characterised from *Alternaria* sp. was alterporriol R (**60**), which was determined to be an isomer of alterporriol Q **59** [49]. These compounds were comprised of two macrosporin (**54**) sub-units. The two sub-units of alterporriol Q (**59**) were linked through carbons C-4 and C-6’, while alterporriol R (**60**) was connected via carbons C-4 and C-8^′^. Alterporriol Q (**59**) exhibited antiviral activity against the porcine reproductive and respiratory syndrome virus (PRRSV), with an IC_50_ value of 39 μM [49]. All the above-mentioned compounds were isolated via a similar protocol in the choice of culture conditions, mobile phase in column chromatography and HPLC purification. The chemical structures of the reported bianthraquinones are shown in Figure 5.

### 4.4. Amino Acid Derivates

**Figure 6:**
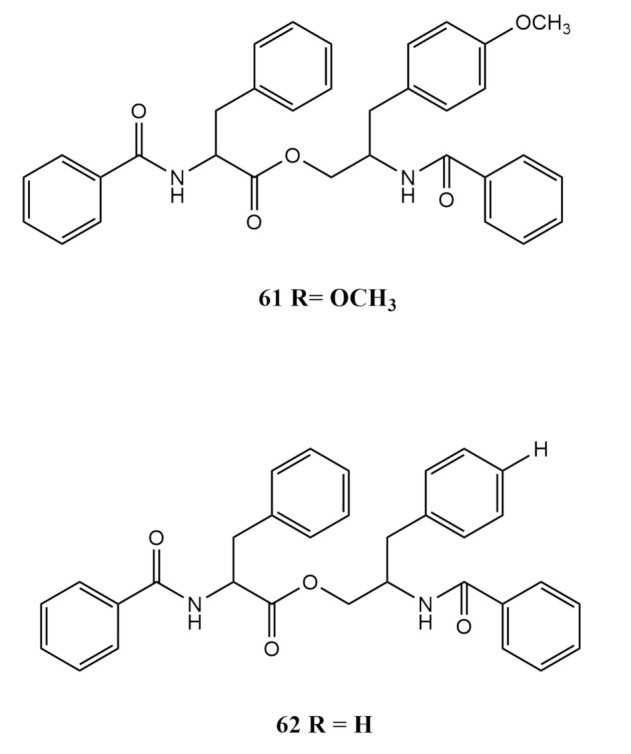
The chemical structures of 4^′^-OMe-asperphenamate (**61**) and asperphenamate (**62**).

In 2013, a phenylalanine derivative 4^′^-OMe-asperphenamate (**61**) was isolated from *Aspergillus elegans* derived from *Sarcophyton* sp. [58]. Asperphenamate (**62**) is another phenylalanine derivative reported from the same study and has an identical basic skeletal structure to the white powdered 4^′^-OMe-asperphenamate [58]. The only difference observed in the ^1^H-NMR spectrum was the presence of a highly electronegative primary methyl signal at δH 3.74 in 4^′^-OMe-asperphenamate (**61**) instead of an aromatic proton at δH 7.30 in asperphenamate (**62**), making it the only detected difference between the two at the carbon position 4^′^ [58]. The compounds were isolated in the same manner as the previously mentioned compounds; column chromatography with Petr Ether and EtOAc mobile phase followed by a Sephadex LH-20 column with chloroform and methanol at a ratio 1:1 and HPLC purification using methanol over a Kromasil C18 preparative column [58]. The chemical structures of compounds (**61**) and (**62**) are shown in Figure 6.

### 4.5. Other Metabolites

**Figure 7:**
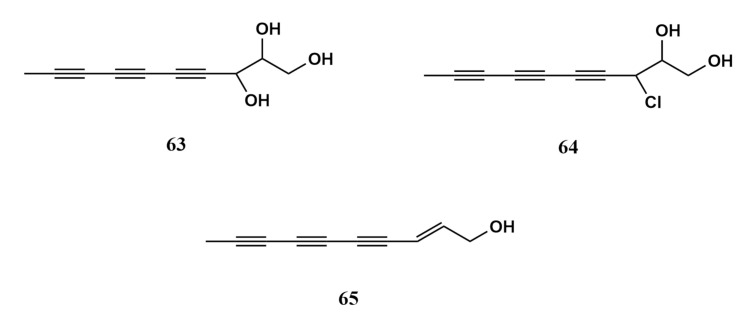
Polyacetylenes (**63–65**) from *Sarcophyton tortuosum*.

In addition to sesquiterpenoid derivates, the marine fungus *Chondrostereum* sp. associated with *Sarcophyton tortuosum* has also produced two novel polyacetylenes, chondrosterins G–H (**63**–**64**), as well as a known polyacetylene (2*E*)-decene-4,6,8-triyn-1-ol (**64**) [68]. Chondrosterin G (**63**) was isolated in the form of a colourless solid. It was structurally determined as deca-4,6,8-triyn-1,2,3-triol [68]. Chondrosterin H (**64**) was reported as a white solid made up of a similar functional group as chondrosterin G (**63**) [68]. Chondrosterin H (**64**) was determined as a 3-chlorodeca-4,6,8-triyn-1,2-diol. Though structurally identical, the difference between chondrosterins G (**63**) and H (**64**) is on the functional group attached to carbon position C-3 where the chlorin atom was observed in chondrosterin H (**64**) instead of a hydroxyl as in chondrosterin G (**63**). The third metabolite from this investigation was (2*E*)-decene-4,6,8-triyn-1-ol (**65**) (a synonym of dehydromatricarianol), which was common in basidiomycete-type fungus [68]. Compounds (**63**–**65**) can be easily but slowly oxidised in air, and this process accelerates with heat. Since (2*E*)-decene-4,6,8-triyn-1-ol (**65**) was the main polyacetylenic metabolite in the investigated fungi, it was proposed as a possible precursor in the biosynthesis for chondrosterins G and H (**63**–**64**) [68]. It was suggested that the double bond of (2*E*)-decene-4,6,8-triyn-1-ol (**65**) be epoxidated and hydrolysed to form a diol as in chondrosterin G (**63**), while Cl^−^ (a nucleophile) together with H^+^ reacts with the epoxidated product to form the halogenated alcohol of chondrosterin H (**64**) [68]. Structures of the polyacetylenes compound **63**–**65** are exhibited in Figure 7.

In 2018, three new compounds were isolated from *Sarcophyton subviride* associated marine fungus *Aspergillus terreus* in the South China Sea. The cooked rice cultured fungus extract yielded luteoride E (**66**), versicolactone G (**67**) and (3*E*,7*E*)-4,8-di-methyl-undecane-3,7-diene-1,11-diol (**68**). Additionally, nine more metabolites comprising of asterrelenin (**69**), methyl 3,4,5-trimethoxy-2-(2-(nicotinamido)benzamido)benzoate (**70**), 14α-hydroxyergosta-4,7,22-triene-3,6-dione (**71**), territrem A (**72**), territrem B (**73**), territrem C (**74**), lovastatin (**75**), monacolin L acid methyl ester (**76**) and monacolin L (**77**) were also characterised [52].

Luteoride E (**66**), a prenylated tryptophan derivative with a 3,7-disubstituted indole was obtained as a yellow oil. Careful interpretation of luteoride A chemical data determined the geometry of the oxime functionality of luteoride E (**66**) to be of *E*-form [52]. Luteoride E (**66**) exhibited inhibitory potency against Lipopolysaccharide (LPS)-induced nitric oxide (NO) production of RAW 264.7 cells with an IC_50_ value of 24.64 μM. The butenolide, versicolactone G (**67**), which was isolated as an amorphous white powder, was characterised to have a basic skeleton made up of a mono-substituted and a trisubstituted benzene identical to a previously reported versilactone B. [52]. The difference between these two metabolites is the presence of a sp^3^ methylene carbon and oxygenated tertiary carbon via a methoxy group in versicolactone G (**67**) instead of the existing δ double bond in versicolactone B [52]. Alongside luteoride E (**66**) and versicolactone G (**67**), (3*E*,7*E*)-4,8-di-methyl-undecane-3,7-diene-1,11-diol (**68**), a linear aliphatic alcohol was isolated as colourless oil and characterised as C_13_H_24_O_2_. When screened for the α-glucosidase inhibitory activity of versicolactone G of the compounds (**66**–**68**), **67** demonstrated potential inhibitory potency with an IC_50_ value of 104.8 ± 9.5 μM [52]. None of these metabolites were antibacterial; however, all three exhibited anti-inflammatory activity against NO production with IC_50_ values between 15.7 and 24.6 μM. Asterrelenin (**69**) is a colourless cubic crystal with infrared peaks detected at 3273, 1691 and 1647 cm^−1^ wavelengths, and carbon chemical shifts at 170.0, 168.7 and 166.4 ppm were indications of amides present [52].

Methyl 3,4,5-trimethoxy-2-(2-(nicotinamido)benzamido)benzoate (**70**) was previously reported from *Aspergillus terreus* cultured under high saline conditions (10% salt) [94]. Salt concentration of 3% and lower did not trigger the production of compound **70**. This compound was mildly antibacterial with a minimum inhibition concentration 52.4 μM against *Staphylococcus aureus* and *Enterobacter aerogenes* [95]. *Aspergillus terreus* also yielded 14α-hydroxyergosta-4,7,22-triene-3,6-dione (**71**), C_28_H_40_O_3_, which was only reported through synthesis prior to its natural isolation from the soft coral fungi [96].

**Figure 8:**
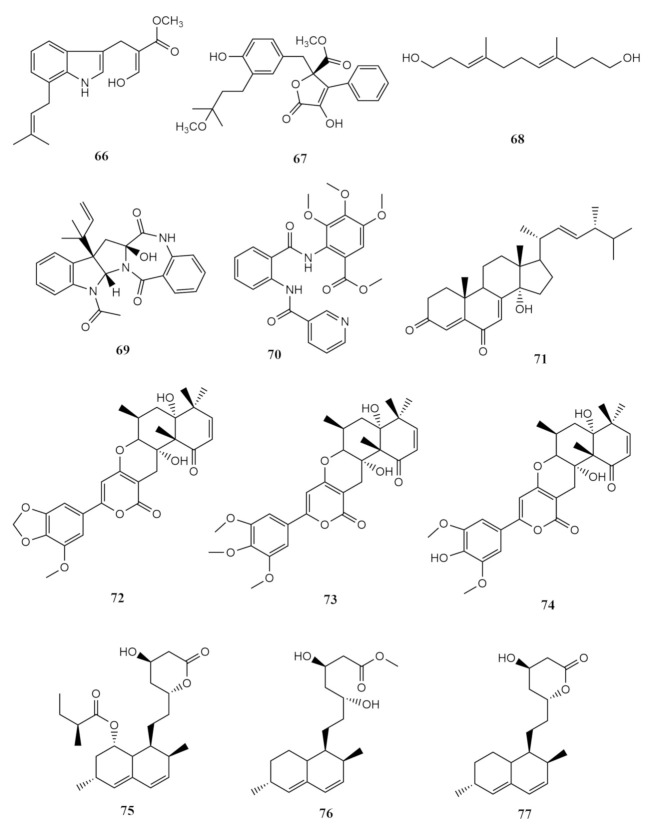
Additional metabolites (**66**–**77**) from the soft-coral *Sarcophyton*-derived marine fungi.

Additionally, three nitrogen lacking termorgenic mycotoxins territrems A–C (**72**–**74**) were successfully characterised from the CHCl_3_ of the fungal strain as well [52]. Previous reports on these compounds were from the *Aspergillus terreus* strain from rice culture. Territrems B and C (**73**–**74**) displayed strong anti-acetylcholinesterase inhibition with IC_50_ values of 4.2 ± 0.6 and 20.1 ± 3.3, respectively [97]. The lovastatin analogue (**75**), C_24_H_36_O_5_, isolated from the soft coral fungi here, is a well-known fungal secondary metabolite previously reported from *Aspergillus sclerotiorum*. Lovastatins are an inhibitor of hydroxymethylglutaryl-coenzyme A reductase (HMGR-CoA). It is also associated with the cause of reduced cholesterol in humans and is cytotoxic to MCF-7, the human cervical cancer cell line (HeLa), the human liver cancer cell line (HepG2), and the human skin melanoma cell line (B16F10). The lovastatin analogue (**75**) exhibited cytotoxicity towards Vero (normal kidney) cells with IC_50_ in the range of 2.2–8.4 μM. It also inhibited the HMGR-CoA activity by 42% at 200 μM [98]. The compounds territrem A (**72**) and lovastatin (**75**) were evaluated for their anti-inflammatory activity against NO production and significant inhibitory potency with IC_50_ values between 5.48 and 29.34 μM was observed [52]. The last two compounds reported from the *Aspergillus terreus* were monacolin L acid methyl ester (**76**) and monacolin L (**77**). These were formerly reported as a byproduct from the fermentation of sterile rice using the fungal strain *Monascus purpureus* to produce a traditional Chinese food and medicine called the red yeast rice [99]. The above described compounds **66**–**77** are shown in Figure 8.

**Figure 9:**
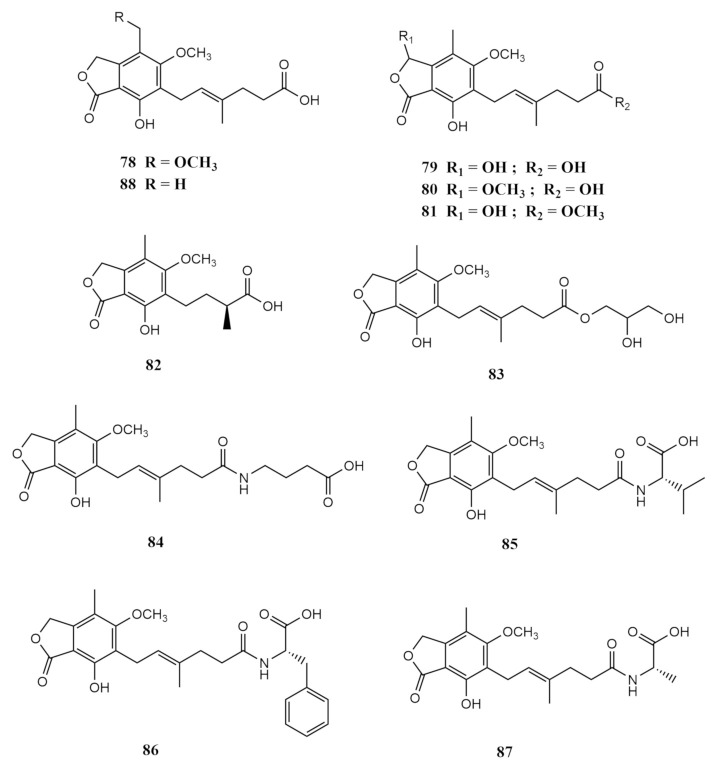
Additional metabolites (**78**–**88**) from the soft-coral *Sarcophyton*-derived marine fungi (*cont.*).

In 2018, a total of 11 acyclic merohemiterpenes were isolated from the *Sarcophyton subviride*-derived fungus *Penicillium bialowiezense* in the South China Sea [53]. The potato dextrose agar (PDA) cultured fungus yielded the compounds 8-O-methyl mycophenolic acid (**78**), 3-hydroxy mycophenolic acid (**79**), 6-(5-carboxy-3-methylpent-2-enyl)-7-hydroxy-3,5-dimethoxy-4-methylphthalan-1-one (**80**), 6-(5-methoxycarbonyl-3-methylpent-2-enyl)-3,7-dihydroxy-5-methoxy-4-methylphthalan-1-one (**81**), 6-(3-carboxybutyl)-7-hydroxy-5-methoxy-4 -methylphthalan-1-one (**82**), 6-[5-(2,3-dihydroxy-1-carboxyglyceride)-3-methylpent-2-enyl]-7-hydroxy-5-methoxy-4 -methylphthalan-1-one (**83**), 6-[5-(1-carboxy-4-*N*-carboxylate)-3-methylpent-2-enyl]-7-hydroxy-5-methoxy-4-methylphthalan-1-one (**84**), *N*-mycophenoyl-l-valine (**85**), *N*-mycophenoyl-l-phenyloalanine (**86**), *N*-mycophenoyl-l-alanine (**87**) and mycophenolic acid (MPA) (**88**) [53]. The crude extract of the cultured fungi was fractioned using column chromatography with the mobile phase combination between petroleum ether: EtOAc: methanol from the ratio 20:1:0 to 1:1:1. The immunosuppressive potential of the fractions exhibited activity at concentrations 1.48 ± 0.15 and 11.83 ± 0.83 μg/mL. Repeated Sephadex column chromatography followed by HPLC over methanol through a Zorbax SB-C18 (9.4 mm × 25 cm) yielded the above-mentioned metabolites [100]. The structures of these compounds are shown in Figure 9.

8-*O*-methyl mycophenolic acid (**78**), 3-hydroxy mycophenolic acid (**79**) and MPA (**88**), being white crystals, are part of the mycophenolic acid family where they are mainly found in the fungal genus *Penicillium*. They are widely known for their diverse bioactivities, such as immunosuppressive and antiviral. Compounds (**80**–**84**) were newly characterised at the time of isolation. Compounds (**80**–**81**) were isolated as white powders [100]. Based on structural elucidation, compound (**80**) was named 6-(5-carboxy-3-methylpent-2-enyl)-7-hydroxy-3,5-dimethoxy-4-methylphthalan-1-one, while compound (**81**) was determined as 6-(5-methoxycarbonyl-3-methylpent-2-enyl)-3,7-dihydroxy-5-methoxy-4-methylphthalan-1-one [100]. 3-hydroxy mycophenolic acid (**79**) and compound (**80**) share identical structures, except that the hydroxyl group at C-3 of (**79**) is replaced by a methoxy in compound (**80**). Comparison between compounds (**80**) and (**81**) shows that the carboxyl group at C-6^′^ in compound (**80**) was methyl-esterified in compound (**81**) [100]. Compound (**82**) was isolated as a white powder and closely resembles euparvic acid. Compound (**83**) was an amorphous white powder known as 2,3-dihydroxypropyl mycophenolate. As for compound (**84**), it was identical to **83** in skeleton, except that the 2,3-dihydroxypropyl group in **83** was replaced by a 4-aminobutanoic acid moiety in **84** [100].

Finally, the compounds *N*-mycophenoyl-l-valine (**85**), *N*-mycophenoyl-l-pheny loalanine (**86**) and *N*-mycophenoyl-l-alanine (**87**) were colourless solids. [53]. Compounds (**77**–**88**) showed inhibitory potency against inosine-50-monophosphate dehydrogenase (IMPDH2) with IC_50_ values between 0.59 and 24.68 μM. When testing their immunosuppressive activity against the proliferation of T-lymphocytes in vitro, the IC_50_ values of compounds (**78**–**80**) were from 0.84 to 0.95 μM, while the IC_50_ values of compounds (**81**–**88**) ranged from 3.27 to 24.68 μM [100].

## 5. Concluding Remarks

The chemical diversity of soft-coral associated symbionts is often limited to bacterial and fungal isolates cultured under laboratory conditions. Nevertheless, there are still many unexplored symbionts in terms of their secondary metabolism and natural-product biosynthesis potential. However, the development of new techniques such as metabolomics for the determination of metabolites produced by specific genes and next generation sequencing creates new dimensions of in-depth investigation of the microbiome. Independent culture methods, such as the next-generation sequencing on sponges, would reveal novel microorganisms, and their guild patterns could be analysed in order to know their association with corals. The role of fungi and their respective host can either be symbiotic or parasitic. As sponges, there have been speculations as to the origin of metabolites isolated from the soft corals. This review study reveals that no common metabolites were shared by the host and its fungi. This confirms that the metabolites isolated from the host are synthesised by the host itself. Due to the diverse bioactivity of the fungal metabolites, we hypothesise that fungal metabolites perform various functions for additional protection to their host, presumably similar to the role of constituents, such as the MAAs. The presence of microorganisms triggers the development of a wide array of secondary metabolites, which function as mutual defences or for adaptive purposes as well as microbial regulation of the octocoral holobionts. Recent studies have shown an increasing trend in bioactive secondary metabolites from *Sarcophyton*-associated marine fungi. However, many compounds have not been thoroughly evaluated for their bioactivities. In the future, more bioassays could be conducted on the soft coral and its associated fungal chemical compounds.

## Figures and Tables

**Table 1:** Sarcophyton species diversity.

Species Name	Region	Reference
*Sarcophyton aalbersbergi* Feussner and Waqa, 2013	Fiji Island	[15]
*Sarcophyton acutum* Tixier-Durivault, 1970	Western Central Pacific	[16]
*Sarcophyton agaricum* (Stimpson, 1855)	Chinese, Japanese Sea	[17]
*Sarcophyton aldersladei* Feussner and Waqa, 2013	Fiji Islands	[15]
*Sarcophyton alexanderi* Feussner and Waqa, 2013	Fiji Islands	[15]
*Sarcophyton auritum* Verseveldt and Benayahu, 1978	Red Sea	[18]
*Sarcophyton birkelandi* Verseveldt, 1978	Micronesian Islands	[19]
*Sarcophyton boettgeri* Schenk, 1896	Indonesia	[14]
*Sarcophyton boletiforme* Tixier-Durivault, 1958	China seas	[20]
*Sarcophyton buitendijki* Verseveldt, 1982	Southern Taiwan	[21]
*Sarcophyton cherbonnieri* Tixier-Durivault, 1958	Madagascar	[22]
*Sarcophyton cinereum* Tixier-Durivault, 1946	Madagascar, Vietnam, Japan	[22,23,24]
*Sarcophyton cornispiculatum* Verseveldt, 1971	Madagascar, China Seas	[20,25]
*Sarcophyton crassocaule* Moser, 1919	Philippines, Madagascar, Vietnam, China seas	[20,22,23,26]
*Sarcophyton crassum* Tixier-Durivault, 1946	Madagascar, New Caledonia, South-West Indian Ocean	[22,27,28]
*Sarcophyton digitatum* Moser, 1919	Philippines, Great Barrier Reef, West-Pacific islands, Madagascar, New Caledonia	[22,23,26,29,30]
*Sarcophyton ehrenbergi* von Marenzeller, 1886	Philippines, Madagascar, Vietnam, China seas	[20,22,23]
*Sarcophyton elegans* Moser, 1919	Philippines, Madagascar, New Caledonia, Hong Kong, Japan, China seas	[20,22,23,24,31,32]
*Sarcophyton expandum* Kolliker	Samoa Islands, South Pacific Ocean	[33]
*Sarcophyton flexuosum* Tixier-Durivault, 1966	Madagascar, South-West Indian Ocean	[22,28]
*Sarcophyton furcatum* Li, 1984	China Seas	[20]
*Sarcophyton gemmatum* Verseveldt and Benayahu, 1978	Red Sea	[18]
*Sarcophyton glaucum* (Quoy and Gaimard, 1833)	Philippines, Madagascar, Red Sea, West-Pacific islands, China seas	[20,22,30,32,34]
*Sarcophyton globoverruccatum* Benayahu and Verseveldt, 1983	Red Sea	[35]
*Sarcophyton griffini* Moser, 1919	Papua New Guinea	[33]
*Sarcophyton infundibuliforme* Tixier-Durivault, 1958	Madagascar, New Caledonia, South-West Indian ocean, China seas	[20,22,23,28]
*Sarcophyton latum* (Dana, 1846)	Philippines, Malay Archipelago, Madagascar, China Seas	[20,22,32,36]
*Sarcophyton mililatensis* Verseveldt and Tursch, 1979	Bismarck Sea	[37]
*Sarcophyton minusculum* Samimi Namin and van Ofwegen, 2009	Persian Gulf	[38]
*Sarcophyton nanwanensis* Benayahu and Perkol-Finkel, 2004	southern Taiwan	[39]
*Sarcophyton nigrum* May, 1899	Marshall Islands, North Pacific Ocean	[33]
*Sarcophyton pauciplicatum* Verseveldt and Benayahu, 1978	Red Sea	[18]
*Sarcophyton portentosum* Tixier-Durivault, 1970	New Caledonia	[22]
*Sarcophyton pulchellum* (Tixier-Durivault, 1957)	Indian Waters, Japan	[33]
*Sarcophyton regulare* Tixier-Durivault, 1946	Madagascar, New Caledonia	[22,23]
*Sarcophyton roseum* Pratt, 1903	Maldives	[40]
*Sarcophyton serenei* Tixier-Durivault, 1958	Vietnam	[23]
*Sarcophyton skeltoni* Feussner and Waqa, 2013	Fiji Islands	[15]
*Sarcophyton soapiae* Feussner and Waqa, 2013	Fiji Islands	[15]
*Sarcophyton solidum* Tixier-Durivault, 1958	Madagascar	[22]
*Sarcophyton spinospiculatum* Alderslade and Shirwaiker, 1991	Laccadive Archipelago	[41]
*Sarcophyton spongiosum* Thomson and Dean, 1931	Malay Archipelago, Madagascar	[22,36]
*Sarcophyton stellatum* Kükenthal, 1911	China Seas	[20]
*Sarcophyton stolidotum* Verseveldt, 1971	Madagascar	[25]
*Sarcophyton subviride* Tixier-Durivault, 1958	Madagascar	[22]
*Sarcophyton tenuispiculatum* Thomson and Dean, 1931	West-Pacific islands, Malay Archipelago, New Caledonia	[23,30,36]
*Sarcophyton tortuosum* Tixier-Durivault, 1946		[33]
*Sarcophyton trocheliophorum* von Marenzeller, 1886	Philippines, West-Pacific islands, Malay Archipelago, Madagascar, New Caledonia, Vietnam, Japan, China seas	[20,22,23,24,30,32,36]
*Sarcophyton tumulosum* Benayahu and van Ofwegen, 2009	Hong Kong	[42]
*Sarcophyton turschi* Verseveldt, 1976	Red Sea	[35]

**Table 2:** Soft coral genus *Sarcophyton* and its associated marine fungi.

Soft Coral Species	Fungi	Reference
*Sarcophyton subviride*	*Aspergillus terreus*	[52]
	*Penicillium bialowiezense*	[53]
*Sarcophyton tortuosum*	*Chondrostereum* sp.	[54,55]
	*Alternaria alternata*	
	*Aspergillus versicolor*	
	*Chaunopycnis* sp.	
	*Cladosporium cladosporioides*	
	*Cladosporium dominicanum*	
	*Cladosporium sphaerospermum*	
	*Didymella* sp.	
	*Hypocrea lixii*	
	*Microsphaeropsis* sp.	
	*Paraconiothyrium cyclothyrioides*	[56]
	*Penicillium citrinum*	
	*Tritirachium* sp.	
	*Penicillium janthinellum*	
	*Penicillium oxalicum*	
	*Phoma putaminum*	
	*Phoma* sp.	
	*Pseudocercospora* sp.	
	*Stagonosporopsis cucurbitacearum*	
	*Talaromyces allahabadensis*	
*Sarcophyton* sp.	*Aspergillus elegans*	[57,58]
	*Pseudallescheria boydii*	[59]

**Table 3:** Bioactivity of soft-coral associated marine fungi.

Soft Coral Species	Fungi	Metabolites	Bioactivities	Reference
*Sarcophyton tortuosum*	*Chondrostereum* sp.	chondrosterin A (**5**)	cytotoxic activities against cancer lines A549, CNE2, and LoVo	[54]
		hirsutanol A (**1**)	potent cytotoxic activities against various cancer cell lines	[67]
		incarnal (**11**)	potent cytotoxic activity against various cancer cell lines	[68]
		chondrosterin J (**14**)	potent cytotoxic activities against the cancer cell lines CNE-1 and CNE-2	[69]
		chondrosterin K (**15**)		
		chondrosterins L (**16**)	significant cytotoxicity against various cancer cell lines in vitro	[70]
		chondrosterins M (**17**)		
*Sarcophyton* sp.	*Pseudallescheria boydii*	3,3^′^-cyclohexylidenebis(1H-indole) (**33**)	significant cytotoxic activity against various cancer cell lines	[59]
	*Aspergillus elegans*	4^′^-OMe-asperphenamate (**61**)	antibacterial activity against *Staphylococcus epidermidis*	[57]
	*Alternaria* sp.	altersolanol B (**50**)	potent inhibitory activity against Gram-negative bacteria	[71]
		altersolanol C (**51**)		
	*Alternaria* sp.	ampelanol (**53**)	mild toxicity against the L5178Y mouse lymphoma cells	[72]
*Sarcophyton subviride*	*Aspergillus terreus*	versicolactone G (**67**)	potent α-glucosidase inhibitory activity	[52]
		luteoride E (**66**)		
		(3E,7E)-4,8-dimethyl-undecane-3,7-diene-1,11-diol (**68**)		
		methyl 3,4,5-trimethoxy-2-(2 -(nicotinamido)benzamido)benzoate (**70**)	significant anti-inflammatory activity against NO production	[52]
		territrem A (**72**)		
		lovastatin (**75**)		
	*Penicillium bialowiezense*	8-*O*-methyl mycophenolic acid (**78**)		
		3-hydroxy mycophenolic acid (**79**)		
		6-(5-carboxy-3-methylpent-2-enyl)-7-hydroxy-3,5-dimethoxy-4-methylphthalan-1-one (**80**)	inhibitory activity against inosine-50-monophosphate dehydrogenase (IMPDH2)	
		6-(5-methoxycarbonyl-3-methylpent-2-enyl)-3,7-dihydroxy-5-methoxy-4-methylphthalan-1-one (**81**)		
		6-(3-carboxybutyl)-7-hydroxy-5-methoxy-4-methylphthalan-1-one (**82**)	and	[53]
		6-[5-(2,3-dihydroxy-l-carboxyglyceride)-3-methylpent-2-enyl]-7-hydroxy-5-methoxy-4-methylphthalan-1-one (**83**)	in vitro immunosuppressive activity against the proliferation of T-lymphocytes	
		6-[5-(1-carboxy-4-*N*-carboxylate)-3-methylpent-2-enyl]-7-hydroxy-5-methoxy-4-methylphthalan-1-one (**84**)		
		*N*-mycophenoyl-l-valine (**85**)		
		*N*-mycophenoyl–l-phenyloalanine (**86**)		
		*N*-mycophenoyl–l-alanine (**87**)		
		mycophenolic acid (MPA) (**88**)		

## Abbreviations

The following abbreviations are used in this manuscript:

UV	Ultra violet
MNP	Marine natural product
MAA	Mycosporin amino acid
Petr Eth	Petroleum Ether
EtOAc	Ethyl Acetate
MeOH	Methanol
RP-HPLC	Reverse Phased High-Performance Liquid Chromatography
HPLC	High-Performance Liquid Chromatography
NMR	Nuclear Magnetic Resonance
ROS	Reactive Oxygen Species
PDB	Potato Dextrose Broth
PDA	Potato Dextrose Agar
MeCN	Acetonitrile
ODS	Octadecylsilyl
A549	Human lung adenocarcinoma cell line
CNE2	Human nasopharyngeal carcinoma cell line
LoVo	Human colon cancer cell line
IC_50_	Half maximal inhibition concentration
EC_50_	Half maximal effective concentration
GPY	Glucose peptone yeast
COSY	Homonuclear correlation spectroscopy
HMBC	Heteronuclear Multiple Bond Correlation
SUNE1	Human Nasopharyngeal carcinoma cell line
MCF-7	Human breast cancer cell line
CNE1	Human nasopharyngeal carcinoma cell line
Bel7402	Human hepatic cancer cell line
KB	Human epidermoid carcinoma cell line
HONE1	Epithelial tumour cell line
GLC-82	Gejiu Lung Carcinoma-82
SMMC-7721	Human hepatocarcinoma cell line
HL7702	Normal human liver cell
GLC82	Human lung adenocarcinoma cell line
HL 7702	Normal Human Liver Cells
HL-60	Myeloid leukemic cell lines
Am-80	RARα Specific Synthetic Retinoid
L5178Y	Mouse lymphoma cells
HCT-116	Human colorectal carcinoma cell line
HeLa	Human cervical cancer cell line
PRRSV	porcine reproductive and respiratory syndrome virus
DEPT	Distortionless enhancement by polarisation transfer
HMBC	Heteronuclear multiple bond correlation
B16F10	Human skin melanoma cell line
HeLa	Human cervical cancer cell line
HepG2	Human liver cancer cell line
B16F10	Human skin melanoma cell line
Vero	Normal kidney cells
HRESIMS	High-Resolution Electrospray Ionisation Mass Spectrometry
CHCl_3_	Chloroform
OH	Hydroxyl
MIC	Minimum inhibition activity
LPS	Lipopolysaccharide
NO	Nitric oxide
HMGR-CoA	Hydroxymethylglutaryl-coenzyme A reductase
MPA	Mycophenolic acid
IMPDH2	inosine-50-monophosphate dehydrogenase
